# Building connections between biomedical sciences and ethics for medical students

**DOI:** 10.1186/s12909-022-03865-y

**Published:** 2022-12-01

**Authors:** Oluwaseun Olaiya, Travis Hyatt, Alwyn Mathew, Shawn Staudaher, Zachary Bachman, Yuan Zhao

**Affiliations:** 1 Department of Primary Care and Clinical Medicine, College of Osteopathic Medicine, Sam Houston State University, Conroe, USA; 2 College of Osteopathic Medicine, Sam Houston State University, Conroe, USA; 3 Department of Psychology and Philosophy, College of Humanities and Social Sciences, Sam Houston State University, Huntsville, USA; 4 Department of Molecular and Cellular Biology, College of Osteopathic Medicine, Sam Houston State University, Conroe, USA

**Keywords:** Medical ethics, Medical education, Curriculum development, Biomedical science

## Abstract

**Background:**

Medical ethics education is crucial for preparing medical students to face ethical situations that can arise in patient care. Instances of ethics being integrated into biomedical science education to build the connection between human science and ethics is limited. The specific aim of this study was to measure student attitudes towards an innovative curriculum design that integrates ethics education directly into a biomedical science course in pre-clinical medical curriculum.

**Methods:**

In this cross-sectional study, three ethics learning modules were designed and built in a biomedical science course in the pre-clinical curriculum. All students of Class of 2024 who were enrolled in the course in 2021 were included in the study. Each module integrated ethics with basic science topics and was delivered with different teaching modalities. The first module used a documentary about a well-known patient with severe combined immunodeficiency disease. The second module was delivered through a clinical scenario on HIV infection. The third module used small group discussion and debate on the topic of blood transfusion. For evaluation, students were asked to self-identify the ethical challenges associated with each module and complete reflective writing to assess their knowledge and attitude. Quantitative and qualitative analyses were conducted on student perceptions of each module.

**Results:**

Likert scale ratings on the usefulness of each module revealed significantly higher ratings for the small group discussion/debate module, seconded by the documentary and lastly the case scenario only modules. Narrative analysis on student feedback revealed three themes: *General favorable impression*, *Perceived learning outcomes*, and *Critiques and suggestion*. Common and unique codes were identified to measure the strengths and weaknesses of each module. Overall, students’ perception of the curriculum design was extremely positive.

**Conclusions:**

This curriculum design enabled us to highlight foundational biomedical sciences and clinical conditions with ethical dilemmas that physicians are likely to face in practice. Students found value in the modules, with a preference for the most active learning method. This study provides insight on a novel approach for integrating medical ethics into biomedical science courses that can be tailored to any institution. Strategies learned include utilizing active learning modalities and discussion.

**Supplementary Information:**

The online version contains supplementary material available at 10.1186/s12909-022-03865-y.

## Background

Medical ethics is the study of the moral issues inherent in the practice of medicine, including, among many other topics, the moral choices physicians face in their day-to-day interactions with patients, colleagues, and the broader society in which they practice [1–4]. Knowledge of medical ethics is crucial for training morally competent healthcare professionals to manage ethical considerations that arise in patient care [5]. Evolving health care systems, expanding involvement of allied health professionals, and advances in technologies and treatment regimens have given rise to increasingly complex moral dilemmas faced by medical professionals in everyday practice. There is thus a compelling argument to continuously improve the incorporation of medical ethics into both pre-clinical and clinical medical education.

In the Association of American Medical Colleges published curriculum report, 143 out of 145 allopathic medical schools covered medical ethics in either a required or an elective course in 2016-2017 academic year [6]. The curriculum topics reported by the American Association of College of Osteopathic Medicine shows all osteopathic medical schools checked off medical ethics in a required course or rotation and 21 out of 38 schools had it covered in a selective/elective course or rotation in academic year of 2017-2018 [7]. Not surprisingly, reports on medical student perspectives of ethics education have revealed strong recognition of the importance of ethics as part of their medical training and a perceived need and desire for more formal bioethical education [8, 9]. Although there is consensus from both faculty and students that medical ethics is an important part of medical training, literature suggests notable heterogeneity across medical schools regarding the best practice of teaching medical ethics [10–12].

Various pedagogical approaches have been employed to teach this subject, including the content, method, and timing of ethics education [10, 13, 14]. In the aspect of curriculum design, ethics inclusion in pre-clinical medical education has been done through various strategies. In addition to the most common traditional stand-alone ethics course, other approaches have also been explored, such as elective courses, students’ medical ethics rounds, a scholarly concentration program, etc [15–18]. Various formats of delivery have been reported as well, including small group session, case-based teaching, narrative approach, peer-based teaching, team-based learning, etc [15, 19–21]. A commonality among these various pedagogical approaches is that the ethics content is delivered in a way that tends to treat ethics as a distinct subject matter that students are required to learn.

A core component of medical education is, of course, also learning the sciences related to understanding the human body. Many of the ethical challenges that doctors face – such as recruiting patients for clinical trials or securing informed consent for an invasive procedure – are directly related to the science that students learn in pre-clinical biomedical education. When an ethics education is cleaved off from the underlying context that gives rise to the ethical issues being studied, it is natural to treat ethics and the sciences core to medicine as inhabiting separate realms: after all, ethics studies how the world *ought* to be while science studies how the world *is*. Ethical norms often become viewed as a set of norms externally imposed on scientists and doctors, rather than norms internal to their practice [22]. But since medicine is fundamentally about using science to treat disease and illness in the context of a doctor-patient relationship, it stands to reason that the aim of the practice of medicine is to use science in a way consistent with the moral norms that govern the doctor-patient relationship. A good doctor, in other words, is one who uses science in an ethical manner to promote healing. Given the way in which ethics and science are interwoven in medical practice, we asked the question whether ethics could be integrated in biomedical science curriculum of pre-clinical medical training. While a review of literature has revealed recent efforts to implement ethics education into science education [23–26], we couldn’t find any discussion of efforts to embed ethics curriculum within the biomedical science curriculum in particular, except for anatomy [27].

Given the rationale above, we initiated a project to develop strategies for medical educators to integrate ethics modules into biomedical science courses, with the aim of promoting student awareness of how scientific practice and ethics are interrelated. Our first step in this project, which this paper analyzes, was to assess student attitudes towards the inclusion of ethics modules in pre-clinical biomedical science courses - how will students respond to this new course design? Future objectives, not undertaken here, will be to measure student learning as a result of our interventions, assess the effectiveness of different inclusion strategies, and create a framework that other medical educators can use in their courses.

Our study concerns a curriculum design we implemented that incorporates ethics threads in a pre-clinical biomedical science course using various teaching modalities. Our model enabled us to highlight the pathophysiology and clinical presentations of the disorders, along with ethical dilemmas that physicians are likely to face in clinical practice. By learning biomedical science side-by-side with medical ethics, students could make meaningful connections between the two domains. We believe this pedagogical approach of teaching medical ethics can help students better understand the relationship between science and ethics in medical practice as well as build richer “organizational structures” of knowledge that will aid in the retention and application of information [28, 29]. This curriculum design can also shed light on how to incorporate ethics education creatively and effectively in the pre-clinical medical curriculum.

## Methods

### Setting

This study was conducted at Sam Houston State University College of Osteopathic Medicine in 2021. Three ethics learning modules were designed and built in a six-week system course “Immune System and HEENT” (HEENT: Head, Eyes, Ears, Nose, Throat) which was offered in the spring semester of the first year of pre-clinical curriculum. In this course, students were introduced to the principles of trauma, inflammatory disorders, infections and cancers associated with HEENT as it relates to the immune system. Students learned to apply the basic concepts of immunology in normal and disease states and to diagnose, prevent, and treat infections, cancers and immunological diseases. All students from our institute who were enrolled in this course in 2021 were included in the study. These students were in their first year of a four-year Doctor of Osteopathic Medicine program. A total of 74 first-year medical students in the Class of 2024 were enrolled in the course and completed all three modules and assessments. Forty were males and thirty-four were females. The average age of the cohort was 26 years ranging from 23 to 45 years.

The learning objectives of the ethics modules were identified and standardized based on the Romanell Report [28] which reviewed medical ethics education in the United States and offers suggestions for objectives, teaching methods, and assessment strategies.

The design of the three modules is presented in Table 1.

**Table 1:** Setting of the three ethics learning modules embedded in biomedical science course

	Activity/Delivery Format	Biomedical Science Topic	Ethics Topic	Assessment (same for all 3 modules)
Module #1	Documentary/Asynchronous	Functions of Immune System	Surrogate Decision Making, Patient Consent and the Interplay of Research and Ethics	1. Identification of ethical challenges associated with each module 2. Completion of narrative responses to reflective questions 3. Completion of a survey question regarding effectiveness of each module
Module #2	Case Only/Hybrid (Synchronous/Asynchronous)	Immunodeficiencies	Beneficence versus Public Health	
Module #3	Case with Small Group Discussion and Debate/Synchronous	Blood Transfusion	Impact of Religion on Clinical Decisions	

The first module used a documentary about David Vetter, a well-known pediatric patient with severe combined immunodeficiency disease. After students completed the session “Introduction of the Immune System”, they were provided an asynchronous ethics module in a learning management software and assigned a one-hour long documentary named *The Boy in the Bubble* released in 2006 by PBS [29], and then completed the assessments at their own time. The second module used a clinical case on human immunodeficiency virus (HIV) that was introduced in team-based learning (TBL), a form of peer collaboration. This case concerned a patient diagnosed with HIV and the dual roles of physician as mandatory reporter of communicable disease and protector of patient confidentiality. Immediately following the two-hour TBL, the students were provided assessments to be completed on their own. The third module was a one-hour mandatory live session offered 4 days after students completed the session “Blood Transfusion”. The students were given an ethics case about a young Jehovah’s Witness in need of a blood transfusion and asked to complete the assessments in class. They were then sorted into small groups for discussion and subsequently assigned a position to debate on whether the patient should receive the blood transfusion. For all three module assessments, students were provided a list of twenty ethical challenges cited from the Romanell report and were asked to select the challenges that they recognized in the learning module and provide supporting explanations (Additional file 1: Appendix 1). Reflective writing prompts were included for students to complete on their own for thinking critically about the ethical challenges associated with the module. Module #1 reflective questions were tied to surrogate decision making and informed consent. An example of the reflective writing prompt from Module #1 includes “Would the case have been handled any differently were David a competent adult? At what point should David be considered autonomous and capable of making healthcare decisions? Explain your reasoning.” Module #2 reflective questions were tied to patient confidentiality and the reporting of communicable diseases. Module #3 reflective questions were tied to the impact of religion on clinical decisions. Students were also asked to voluntarily respond to the perception question “How useful did you consider this module in ethics training?” to rate the usefulness of the module on a 1 to 5 Likert scale (1-not useful at all, 5-very useful) and provide feedback. We expected students took 30 min to 1 h to complete all assessments. General feedback were provided by YZ and OO in person or in writing for each module.

### Analytical procedure

The analytical procedure was aligned with the study’s aim to measure student attitudes about the ethics modules. The first analysis measured differences between perceived usefulness of the modules to determine if students found one teaching modality more useful than the others. The second was a qualitative study on written student feedback.

The statistical analysis of perceived usefulness was performed with the python programming language using the pandas, statsmodels, scipy, and scikit_posthocs packages. Descriptive statistics were calculated for each analysis with reported averages following the format of the mean ± one standard deviation. Group differences between the Likert-based usefulness ratings were initially analyzed with an ANOVA and normalcy of the standardized residuals were computed with a Shapiro-Wilk test. The final analysis used a Kruskal-Wallis test and post-hoc Dunn test with a Bonferroni correction to determine differences between groups (corrected-*α* for all tests was set to 0.05).

Student feedback was analyzed using two different qualitative approaches: constant comparison analysis [30] and classical content analysis [31]. Using more than one approach in qualitative data analysis, as recommended by Leech and Onwuegbuzie [32], can increase interpretive validity, or the degree to which the perspectives of students are accurately rendered by the researcher [33]. Two of the investigators (YZ and KO) double coded the de-identified student feedback with Dedoose 8.3.47b to independently assign codes to the text for each module. The investigators then reviewed the accuracy and relevance of these codes according to their interpretation of the students’ meaning and used the software to merge similar codes and remove other codes that were no longer pertinent. Next, the investigators used printouts from the software to complete axial coding, which involves comparing text segments and codes to create categories made up of similar codes, and to combine categories into broad themes. Last, the investigators used printouts from the software to conduct classical content analysis, calculating percentages of codes associated with each theme to determine their relative significance to the participants. The premise underlying classical content analysis is that the frequency of occurrence is connected to the meaning of the content [31]. This analysis allowed the investigators to discover the relative importance that each theme held for students (i.e., based upon the frequency of the codes associated with each theme), which gave more insight into students’ responses. The data were entered into Microsoft Excel for data management.

### Ethical considerations

Exempt status for the research project was granted by the IRB committee of SHSU.

## Results

**Fig. 1:**
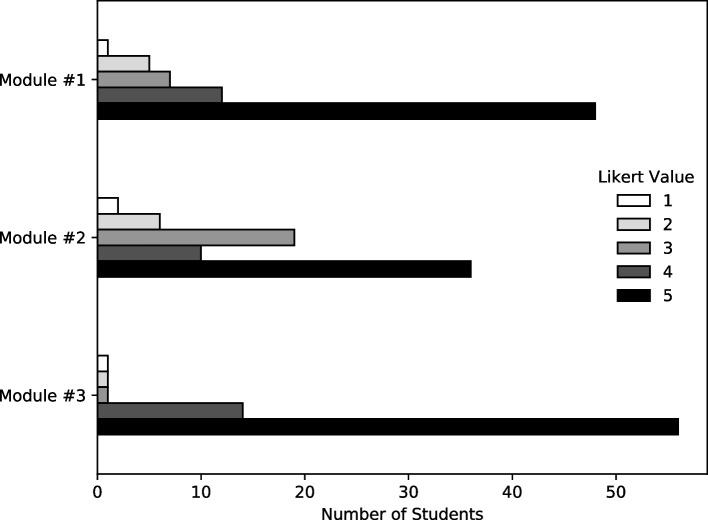
Usefulness ratings for each ethics learning module

The majority of students (73/74) completed the Likert-based usefulness ratings. In general, students found each module useful, with an average across all modules of 4.37 ± 0.99. Descriptive statistics for each module are reported in Table 2 and the distribution of answers are shown in Fig. 1.

**Table 2:** Descriptive statistics of usefulness Likert ratings

	Count	Mean	SD	Min	Max
Module #1	73	4.4	1	1	5
Module #2	73	4.0	1	1	5
Module #3	73	4.7	0.7	1	5

To test differences between the Likert-based usefulness ratings between modules, a one-way ANOVA was performed with modules as groups and Likert-results as the dependent variable. However, it was found that the standardized residuals of the ANOVA did not follow a normal distribution after testing with a Shapiro-Wilk test (*W* = 0.84, *p* < 0.001). Due to non-normal standardized residuals, a Kruskal-Wallis test was employed and found a statistically significant difference in rank-order between treatments (*H* = 16.2, *p* < 0.001). A post-hoc Dunn test with a Bonferroni correction found that the only treatment pair with a statistically significant difference (corrected-*α* < 0.05) was between Module #3 and Module #2 (corrected *p* < 0.001). Complete results from the post-hoc Dunn test are reported in Table 3.

**Table 3:** Post-hoc Dunn test for usefulness Likert ratings

	Comparison Module	*p*
Module #1	Module #2	0.053
Module #1	Module #3	0.27
Module #2	Module #3	< 0.001***

The number of narrative responses to the perception question was consistently high, but not complete, with 82% of students who completed Module #1 providing feedback, with 85% for Module #2 and 80% for Module #3. Constant comparison analysis of student perception of the learning modules reveals three themes. These include *general favorable impression for the learning modules*, *perceived learning outcomes for the learning module*, and *suggestions and critiques from students *(Table 4).

**Table 4:** Themes identified via constant comparison analysis in order of importance

Themes	Codes	Frequency of Codes	Percent of Codes
General Favorable Impression on Ethics Learning Modules	Engaging and enjoyable	194	52.43
	Effective		
	Thought provoking		
	Challenging		
	Discussion provided opportunity to learn from each other		
	Provided opportunity to present and view different perspectives		
	Loved format of debate		
	Exposure to different viewpoints		
	Better than reading		
	Efficient use of time		
	Stress-free activity		
Perceived Learning Outcomes for Ethics Learning Modules	Real world preparation and increased awareness of their role as future physician	140	37.84
	Raised awareness of the complexity of ethics		
	Built connection of ethics and science		
	Raised awareness of the interplay between ethics and law		
	Raised awareness of connection of ethics and human research		
	Increased awareness of complexity of patient care		
	Helped understand the role of ethics in health care		
	Increases awareness of the role of religion in health care		
Suggestions and Critiques for Ethics Learning Modules	Case being hypothetical with lack of background information	36	9.73
	Wished for more structural instruction methods		
	Prefer discussing in groups		
	Time consuming		
	Prefer free flow refection		
	Prefer documentary		
	Need time to consider		
Total			100

### General favorable impression of students for the learning modules

Students’ overall impression of the ethics learning modules integrated in a biomedical science course was positive. Based on classical content analysis (Table 4), the student’s general impression theme contains the highest percentage of codes, suggesting it is the most relevant theme from students’ perceptive responses. A detailed breakdown of common and unique codes for this theme is presented in Table 5.

**Table 5:** Frequency of codes of theme one: general favorable impression on ethics learning modules

	Module #1	Module #2	Module #3
Common Codes			
Engaging and enjoyable	24	9	21
Effective	19	10	23
Thought provoking	7	13	18
Unique Codes			
Exposure to different viewpoints	4	–	–
Better than reading	2	–	–
Challenging	–	–	12
Discussion provided opportunity to learn from each other	–	–	10
Provided opportunity to present and view different perspectives	–	–	11
Loved format of debate	–	–	9
Efficient use of time	–	–	1
Stress-free activity	–	–	1

*Engaging and enjoyable* is the most dominant code in this theme with more comments from Module #1 (documentary) and Module #3 (SGD/Debate). In addition, several students described participating in Module #3 as “*fun*”.

*“It was very interesting to learn about ethics this way and certainly something that I will not forget for a very long time.”* (Module #1)

*“Everyone in my group was excited to participate and contribute thought. I loved this.”* (Module #3)

According to students, all the modules were considered effective and useful, thought provoking, and provided opportunities for them to examine ethical challenges and different perspectives which promoted their critical thinking. Most of the relevant comments associated with these codes were from Module #3, seconded by Module #2 and then Module #1.

*“If I had watched the documentary on my own, I probably would not have thought about it as deeply as I did for this activity”* (Module #1)

*“ … the questions challenge me to think from different perspectives and consider multiples factors.”* (Module #2)

*“The debate made me think of the case on a deeper level and truly analyze each argument.”* (Module #3)

Unique codes were also identified for Modules #1 and #3. For Module #1, students commented that watching the documentary helped them to see different viewpoints and it is more effective than traditional teaching styles such as reading text. For Module #3, the students described the debate as stress free but challenging and highlighted that it provided the opportunity to present and view different perspectives which ultimately allowed them to learn from each other. It was well perceived by students as a favorable format of teaching ethics.

*“Thus, having these discussions are still very important, and sharing unique perspectives is great for that in two regards. One, these discussions teach us who others are and what others think about the world around them, and we must try our best to respect and understand these perspectives of others. Two, these discussions could reveal more about ourselves and even help us understand ourselves better, which allows us to develop our sense of uniqueness.”* (Module #3)

### Perceived learning outcome

Our analysis also revealed students’ perceived learning outcomes for each module. Several common and overlapping codes were identified as well as unique codes. (Table 6).

**Table 6:** Frequency of codes of theme two: perceived learning outcomes for ethics learning modules

	Module #1	Module #2	Module #3
Common Codes			
Raised awareness of the complexity of ethics	14	7	5
Real world preparation and increased awareness of their role as future physician	13	24	31
Built connection of ethics and science	9	5	1
Unique Codes			
Raised awareness of connection of ethics and human research	7	–	–
Promoted critical thinking	4	–	–
Helped understand the role of ethics in health care	3	–	–
Raised awareness of the interplay between ethics and law	–	10	–
Increased awareness of complexity of patient care	–	–	4
Increases awareness of the role of religion in health care	–	–	3

Many students felt that all the modules provided real world preparation and increased awareness of their roles as future physician. This code was the most dominant one compared to the other common codes. They felt that the modules helped them recognize the impact of ethical issues in clinical situations and made them think ahead as to how they might and should proceed in real-life circumstances.

*“ … this is a very ethically engaging case and an issue that we will likely come across in our careers.”* (Module #1)

*“Really challenging situations like this do happen in real life and we need to have the skills to navigate through these situations and do what is best for the patient and their life.”* (Module #3)

Students also described that the modules helped them raise awareness of the complexity of ethics by *seeing the difficulty of ethics and how sometimes there is not a clear-cut answer as to what to do in a situation*. In addition, the integration of ethics learning in biomedical science course helped them build connection of ethics with science. The classroom activities encouraged the application of biomedical knowledge learned in the course.

*“I found this module to be useful in terms of utilizing all that we have learned so far to understand HIV from a different lens than previously thought.”* (Module #2)

*“I really like seeing the ethical side of the science that we are learning. It is easy to get so focused in the science and technology that it is nice to take a step back and think of the human perspective of it.”* (Module #3)

The unique codes for the perceived learning outcomes were consistent with the distinct ethical challenges that were highlighted in each module. In Module #1, several students felt it improved awareness of the connection of ethics and research as well as recognizing the importance of a research compliance body oversight. Students felt this module helped them understand the role of ethics within the larger health care system. One student commented:

*“My value of the scientific community and of institutional review boards has now increased as I believe that they could have helped improve the situation David and his family were facing if they intervened appropriately.*” (Module #1)

In Module #2, many students felt it raised awareness of the interplay between ethics and law, *made them consider the legal rights* versus *the patients’ rights* when it comes down to certain situations as physician.In Module #3, students identified increased awareness of the complexity of patient care as well and of the role of religion in health care. They also felt this highlighted the importance of patient-centered care.

*“ … physicians must not just deal with symptoms but also the social aspects and ethical principles when addressing a patient’s care. Education, personal experience, stress, and religious beliefs are a few of the variables that differ amongst individuals and increase the complexity of a patient case.*” (Module #3)

### Student critiques and suggestions

Some students commented on the fact that addition of group discussion would have been preferred and more effective in both Module #1 and #2. In Module #1, some wished for more structured instruction *along with concrete objectives and didactic information*. In Module #2, some students felt that the case was hypothetical and lacked background information. As one student commented “*It would be more useful knowing more about the state laws and regulations surrounding this kind of diagnosis.”* Adding more context to the case and providing relevant learning materials *would allow for more insightful discussion to the suggested way to approach difficult scenarios for us as future physicians*. In Module #3, one student felt they needed more time to consider the ethical challenges as *it was a harder ethics choice*.

Although the goal is for students to explore and identify ethical challenges on their own, one student commented the Module #1 is *not instructional in pointing out ethical issues/errors in the video as they happen.* A detailed breakdown of common and unique codes for this theme is presented in Table 7.

**Table 7:** Frequency of codes of theme three: suggestions and critiques from students for ethics learning modules

	Module #1	Module #2	Module #3
Wish for more structural instruction methods	6	–	1
Time consuming	4	1	–
Prefer discussing in groups	4	3	–
Prefer free flow reflection	2	–	–
Case being hypothetical with lack of background information	–	14	–
Prefer documentary	–	2	–
Need time to consider	–	–	1

## Discussion

Given the importance of ethics in medical education, we created an innovative curriculum design for ethics learning made up of three unique modules that were integrated into a biomedical science course in the first-year pre-clinical curriculum. We started this project with the overall aim to increase student awareness and understanding of the ethical dimensions of the biomedical sciences. The literature on interleaving would suggest that students who learn medical ethics within a biomedical science context will improve their learning of both the foundational science content and the medical ethics content [34], for by exercising different forms of reasoning – scientific reasoning and ethical reasoning – within the same course, students may increase their ability to retain and apply the content learned, at least as compared to massed learning [35]. Literature is limited regarding strategies to integrate ethics in biomedical science courses [35]. Ultimately, we believe that a curricular design like the one that we developed can help medical students build connections between science, human disease and ethics, but our first step for this project was to see how students would react to this novel course design by evaluating their attitudes.

The design of our ethics modules was heavily influenced by the mounting evidence suggesting that students learn better and retain information longer when they learn through multiple modalities [35]. Several educational modalities have been shown to be effective in the teaching and learning of ethics in medical education. Examples include the use of ethical dilemmas in integrated small group sessions, standardized patients, team-based approaches, case-based discussion, problem-based methods, student-driven curriculum, peer-based teaching and ethics guest lectures [4, 10, 13, 20, 36, 37]. These teaching modalities additionally provide opportunities for active learning which can increase student engagement and retention of information [35].

With this in mind, our modules were created utilizing different modalities to allow for maximal engagement and connection with the content. The particular choice of active learning strategy for each module was made by considering the content and the availability of course schedule along with the instructors’ content expertise. All three modules generated a consensus regarding the effectiveness and benefits of this curriculum design of ethics education in improving understanding and future preparation for encountering real dilemmas in medical practice. While all modules were considered to be engaging and thought-provoking, student responses highlighted various perceived strengths and weaknesses of each unique module and pedagogical modality. Module #1 was delivered through an asynchronous module using a commercially available documentary without formalized discussion. While the design of the documentary module did not allow for collaboration between the students or didactic instruction, choosing media with an existing reputation for engaging audiences made it more likely that the students would have at least a base-level interest in the module. Interactive learning strategies such as using the documentary as a basis for an interrupted case study could be utilized in the future to enhance the engagement. Module #2 was presented in a case-based fashion and without group discussion. A perceived weakness of this module was the lack of detailed background information in comparison to Module #1 which is a well-publicized case with robust details. Since the module was embedded in a TBL case that was focused on the scientific foundations of HIV, students felt it helped them strengthen their understanding of the ethical dimensions of the science they learned. Module #3 allowed for both small and large group discussion while incorporating a debate format which prompted rich discussion.

Although all modules were considered useful, student responses indicated a strong preference for Module #3, with a statistical significance when compared to Module #2, but not Module #1. There were more unique codes and comments generated related to its complexity and challenging format. This could be because the debate allowed students the uninterrupted opportunity to voice an opinion regarding the many ethical dilemmas central to the case being examined. Further, students enjoyed learning about their classmates and hearing new viewpoints from colleagues. Students were assigned a side to defend which compelled some students to make arguments different from their own perspective. Our finding resonates with existing literature which has suggested that the use of debates can be an effective tool for teaching medical ethics because it increases students’ critical thinking expression and tolerance toward ambiguity [38]. In addition, the reflective writing time was integrated into the session module which encouraged more valuable, thorough, and accurate feedback. Another reason students may have reported a preference for Module #3 could be that it was the last module of the course and close to the completion of the course. Overall, these elements highlighted the benefit of a debate format to encourage discussion of difficult topics emphasized in ethics courses, which contributed to the preference of Module #3. Interestingly, only one participant mentioned the link between ethics and science for Module #3, this might be due to the timing and method of the science session delivery. The session “Blood Transfusion” was offered asynchronously at the beginning of the week, while Module #3 was delivered at the end of the week due to scheduling conflict. This suggests the importance of purposeful design, delivery, and sequencing of both science and ethics sessions to help students better recognize the connection between the two subjects.

Our study has several limitations that affect the reliability and validity of the study. Although students were provided opportunities to practice ethical reasoning and decision making through providing explanation for self-identified ethical challenges and reflective writing, the direct learning outcome was not assessed. The lack of baseline data has hindered the analysis on the gain of students’ knowledge and attitude, although as a whole they perceived the modules as valuable and beneficial. Future studies should include pre- and post-assessment and longitudinal evaluation of the growth of the knowledge and moral attitudes of students. We also do not know whether students’ usefulness ratings were based on their preference for learning modalities or their specific interest in the topic of the module. For future studies the usefulness question should be revised to remove this ambiguity and improve content validity. The students were also not asked to directly compare the modules. Instead, they gave their responses at the time they completed each module, which was weeks apart from one another. Their general opinion may have changed over time and the order in which the modules were delivered may have influenced their responses. The modules could also be expanded to include multiple classes and to incorporate the modules in multiple courses. Furthermore, backward design strategy could be incorporated to ensure achievement of ethics learning objectives. The long-term impact of the modules may be evaluated by using preceptors survey in clerkship.

Expanding the study, and ethics education in general, faces several obstacles. Perhaps the most challenging obstacles are mundane: the lack of time within curriculum, lack of time in faculty schedules, and the lack of teachers qualified to teach ethics in the context of medical education [10]. Our study shows that ethics may be integrated in non-traditional places in curriculum and that student-directed learning can be used to alleviate the burden of curriculum load, although more student interaction should be encouraged. We plan to develop pre- and post-testing along with additional modules in order to measure longitudinal learning and to further integrate ethics into our biomedical science curriculum. To address the lack of standardized ethics training or certification for the instructors some institutions may face, collaborating with ethicists through interdepartmental or interinstitutional effort may be helpful. Together, the team can develop the modules as well as provide narrative feedback to students, which may enhance the delivery and assessment of the ethics modules.

## Conclusions

Our study demonstrates that ethics education can be integrated with biomedical sciences. As is universal in education, the pedagogical design of the curriculum and relevant activities is the key to gaining students’ interest in learning. Strategies for ethics learning that we noted include the importance of purposeful design and sequence as well as the use of active learning modalities that involve discussion such as debate. Our model can shed light on an innovative way of integrating ethics education into medical education.

## Supplementary Information

**Additional file 1.** Appendix 1. List of Ethical Challenges Cited from the Romanell Report.

## Data Availability

The datasets used and/or analyzed during the current study are available from the corresponding author on reasonable request.

## References

[1] Veatch RM (1997). Medical Ethics.

[2] Beauchamp TL, Childress JF (2013). Principles of biomedical ethics.

[3] Vaugh L (2020). Bioethics: principles, issues, and cases.

[4] Miles SH, Lane LW, Bickel J, Walker RM, Cassel CK (1989). Medical ethics education: coming of age. Acad Med.

[5] Savulescu J, Crisp R, Fulford KWM, Hope T (1999). Evaluating ethics competence in medical education. J Med Ethics.

[6] Curriculum Topics in Required and Elective Courses at Medical School Programs. AAMC. https://www.aamc.org/data-reports/curriculum-reports/interactive-data/curriculum-topics-required-and-elective-courses-medical-school-programs. Accessed 29 Nov 2022.

[7] 2017-18 Osteopathic Medical College Curriculum Topics. AACOM. https://www.aacom.org/reports-programs-initiatives/aacom-reports/curriculum. Accessed 29 Nov 2022.

[8] DeFoor MT, Chung Y, Zadinsky JK, Dowling J, Sams RW (2020). An interprofessional cohort analysis of student interest in medical ethics education: a survey-based quantitative study. BMC Med Ethics.

[9] AlMahmoud T, Hashim MJ, Elzubeir MA, Branicki F (2017). Ethics teaching in a medical education environment: preferences for diversity of learning and assessment methods. Med Educ Online.

[10] Soleymani Lehmann L, Kasoff WS, Koch P, Federman DD (2004). A survey of medical ethics education at U.S. and Canadian medical schools. Acad Med.

[11] DuBois JM, Burkemper J (2002). Ethics education in U.S. medical schools: a study of syllabi. Acad Med.

[12] Shamim M, Baig L, Zubairi N, Torda A (2019). Review of ethics teaching in undergraduate medical education. J Pak Med Assoc..

[13] de la Garza S, Phuoc V, Throneberry S, Blumenthal-Barby J, McCullough L, Coverdale J (2017). Teaching medical ethics in graduate and undergraduate medical education: a systematic review of effectiveness. Acad Psychiatry.

[14] Giubilini A, Milnes S, Savulescu J (2016). The medical ethics curriculum in medical schools: present and future. J Clin Ethics.

[15] Goldie J (2000). Review of ethics curricula in undergraduate medical education. Med Educ.

[16] Aguilera ML, Martínez Siekavizza S, Barchi F (2019). A practical approach to clinical ethics education for undergraduate medical students: a case study from Guatemala. J Med Educ Curric Dev.

[17] Beigy M, Pishgahi G, Moghaddas F, Maghbouli N, Shirbache K, Asghari F (2016). Students’ medical ethics rounds: a combinatorial program for medical ethics education. J Med Ethics Hist Med.

[18] Liu EY, Batten JN, Merrell SB, Shafer A (2018). The long-term impact of a comprehensive scholarly concentration program in biomedical ethics and medical humanities. BMC Med Educ.

[19] Chung EK, Rhee JA, Baik YH, A OS. (2009). The effect of team-based learning in medical ethics education. Med Teach.

[20] Hindmarch T, Allikmets S, Knights F (2015). A narrative review of undergraduate peer-based healthcare ethics teaching. Int J Med Educ.

[21] Donaldson TM, Fistein E, Dunn M (2010). Case-based seminars in medical ethics education: how medical students define and discuss moral problems. J Med Ethics.

[22] Wolpe PR (2006). Reasons scientists avoid thinking about ethics. Cell.

[23] Mcgowan A (2013). Teaching science and ethics to undergraduates: a multidisciplinary approach. Sci Eng Ethics..

[24] Reese AJ (2020). An undergraduate elective course that introduces topics of diversity, equity, and inclusion into discussions of science. J Microbiol Biol Educ.

[25] Mann MK, Kloepper KD, Crawford GL (2017). The right place and the right time: incorporating ethics into the undergraduate biochemistry curriculum. ACS symposium series.

[26] Smith K, Wueste D, Frugoli J (2007). Using “ethics labs” to set a framework for ethical discussion in an undergraduate science course. Biochem Mol Biol Educ.

[27] Cornwall J, Hildebrandt S (2019). Anatomy, education, and ethics in a changing world. Anat Sci Educ.

[28] Carrese JA, Malek J, Watson K, Lehmann LS, Green MJ, McCullough LB (2015). The essential role of medical ethics education in achieving professionalism: the Romanell report. Acad Med.

[29] The boy in the bubble. Public Broadcasting Service (PBS); 2006.

[30] Glaser B (1965). The constant comparative method of qualitative analysis. Soc Probl.

[31] Berelson B (1952). Content analysis in communication research. Ann Am Acad Pol Soc Sci.

[32] Leech NL, Onwuegbuzie AJ (2007). An array of qualitative data analysis tools: a call for data analysis triangulation. Sch Psychol Q.

[33] Maxwell JA (2022). Understanding and validity in qualitative research. The qualitative Researcher’s companion.

[34] Safuan S, Ali S, Kuan G, Long I, Nik N (2017). The challenges of bioethics teaching to mixed-ability classes of health sciences students. Educ Med J.

[35] Brown PC (2014). Make it stick : the science of successful learning.

[36] Mattick K (2006). Teaching and assessing medical ethics: where are we now?. J Med Ethics.

[37] Sullivan BT, DeFoor MT, Hwang B, Flowers WJ, Strong W (2020). A novel peer-directed curriculum to enhance medical ethics training for medical students: a single-institution experience. J Med Educ Curric Dev.

[38] Amar-Gavrilman N, Bentwich ME (2022). To debate or not to debate? Examining the contribution of debating when studying medical ethics in small groups. BMC Med Educ.

