# Supplementary material for: Building connections between biomedical sciences and ethics for medical students
Source: BMC Med Educ. 2022 Dec 1;22:829. doi: 10.1186/s12909-022-03865-y (PMC9714188; doi:10.1186/s12909-022-03865-y)
Supplement: Supplementary file 1 — Additional file 1. Appendix 1. List of Ethical Challenges Cited from the Romanell Report. [file 12909_2022_3865_MOESM1_ESM.docx]

Appendix 1. List of Ethical Challenges Cited from the Romanell Report

| Ethical Challenges | What ethical challenges have you been able to recognize in this documentary? (put a check mark for recognized challenge) | Provide explanation for why you selected this challenge based on the documentary |
| --- | --- | --- |
| Protection of patient privacy and confidentiality |  |  |
| Disclosure of information to patients, including medical errors and the delivery of bad news |  |  |
| Assessment of patient decision-making capacity and issues related to surrogate decision making |  |  |
| Shared decision making, including informed consent and informed refusal of medical interventions by patients |  |  |
| Care at the end of life, including patient advance directives, withholding and withdrawing life-sustaining interventions, care for the dying, and determination of death |  |  |
| Maternal–fetal medicine, including reproductive technologies and termination of pregnancy |  |  |
| Pediatric and neonatal medicine |  |  |
| Access to health care, including health care disparities, the health care system, and the allocation of scarce resources |  |  |
| Cross-cultural communication, including cultural competency and humility |  |  |
| Role of the health care professional’s personal values in the clinical encounter, including the extent and limits of the right of conscience |  |  |
| Conflicts of interest and of obligation in education, clinical practice, and research |  |  |
| Research with human subjects, including institutional review boards |  |  |
| Work within the medical team, including interprofessional interactions |  |  |
| Concerns about colleagues, including impairment, incompetence, and mistakes |  |  |
| Medical trainee issues, including disclosure of student status, the tension between education and best care for patients, the hidden curriculum, and moral distress |  |  |
| Self-awareness, including professional identity and self-care |  |  |
| Management of challenging patients/family members, including recognition of what the clinician may be contributing to the difficulty |  |  |
| Social media |  |  |
| Religion and spirituality |  |  |
| Acceptance of gifts from patients, including grateful patient philanthropy |  |  |
